# Mate-guarding behavior enhances male reproductive success via familiarization with mating partners in medaka fish

**DOI:** 10.1186/s12983-016-0152-2

**Published:** 2016-06-02

**Authors:** Saori Yokoi, Satoshi Ansai, Masato Kinoshita, Kiyoshi Naruse, Yasuhiro Kamei, Larry J. Young, Teruhiro Okuyama, Hideaki Takeuchi

**Affiliations:** Department of Biological Sciences, Graduate School of Science, The University of Tokyo, Tokyo, Japan; Laboratory of Bioresources, National Institute for Basic Biology, Okazaki, Aichi Japan; Division of Applied Biosciences, Graduate School of Agriculture, Kyoto University, Kyoto, Japan; Center for Translational Social Neuroscience, Department of Psychiatry and Behavioral Sciences, Yerkes National Primate Research Center, Emory University, Atlanta, USA; The Picower Institute for Learning and Memory, Massachusetts Institute of Technology, Cambridge, USA; Graduate School of Natural Science and Technology, Okayama University, Okayama, Japan

**Keywords:** Mate-guarding, Familiarity, Female preference, Medaka fish

## Abstract

**Background:**

Male-male competition and female mating preference are major mechanisms of sexual selection, which influences individual fitness. How male-male competition affects female preference, however, remains poorly understood. Under laboratory conditions, medaka (*Oryzias latipes*) males compete to position themselves between a rival male and the female (mate-guarding) in triadic relationships (male, male, and female). In addition, females prefer to mate with visually familiar males. In the present study, to examine whether mate-guarding affects female preference via visual familiarization, we established a novel behavioral test to simultaneously quantify visual familiarization of focal males with females and mate-guarding against rival males. In addition, we investigated the effect of familiarization on male reproductive success in triadic relationships.

**Results:**

Three fish (female, male, male) were placed separately in a transparent three-chamber tank, which allowed the male in the center (near male) to maintain closer proximity to the female than the other male (far male). Placement of the wild-type male in the center blocked visual familiarization of the far male by the female via mate-guarding. In contrast, placement of an arginine-vasotocin receptor mutant male, which exhibits mate-guarding deficits, in the center, allowing for maintaining close proximity to the female, did not block familiarization of the far male by the female. We also demonstrated that the reproductive success of males was significantly decreased by depriving females visual familiarization with the males.

**Conclusions:**

Our findings indicated that, at least in triadic relationships, dominance in mate-guarding, not simply close proximity, allows males to gain familiarity with the female over their rivals, which may enhance female preference for the dominant male. These findings focusing on the triadic relationships of medaka may contribute to our understanding of the adaptive significance of persistent mate-guarding, as well as female preference for familiar mates.

**Electronic supplementary material:**

The online version of this article (doi:10.1186/s12983-016-0152-2) contains supplementary material, which is available to authorized users.

## Background

For successful production of offspring, it is important for males of many animal species to outcompete other males (male-male competition) and be selected by females as their mating partner (female mate choice) [1]. Male-male competition and female mate choice are considered to be major constituents of mating strategies and many studies of these behaviors have been performed individually. For example, social dominance in male-male competition prominently increases reproductive success in many animals, such as zebrafish [2], tropical mockingbirds [3], and macaques [4]. In addition, the innate criteria for female choice of mating partners differ among species. For example, feather length in the long-tailed widowbird [5], body colour pattern in the guppy [6], and the courtship song in the cricket [7] are criteria for mate choice. The interaction between male-male competition and female mate choice, however, remains poorly understood. To address this issue, we focused on mate-guarding behavior in triadic relationships (male, male, and female). Mate-guarding is the behavioral process of maintaining close proximity to a (potential) mating partner to prevent rivals from mating with it [8–13]. As mate-guarding involves triadic relationships, including both male-male interactions and male-female interactions,, we consider that this triadic relationship allow us to study the effects of male-male competition to female mate choice.

In the present study, we used medaka fish (*Oryzias latipes*), which robustly exhibit mate-guarding behavior under laboratory conditions [14]. In triadic relationships (male, male, female), medaka males maintain their position between the female and the rival male without aggressive behavior. As sexually mature medaka female have a short reproductive cycle (24-h) and spawn eggs once each morning [15–17], medaka fish is an interesting model for the study of  mating-related behavior. Medaka males exhibit this mate-guarding irrespective of the mating period, and mate-guarding dominance outside of the mating period positively correlates with male mating success [14]. Generally, persistent mate-guarding is thought to have a high energy cost, which would reduce male survival rate [18], and it therefore remains an open question whether there is some benefit of the persistent mate-guarding, such as enhancement of male reproductive success, in medaka fish.

In addition, medaka females discriminate conspecific males by visually-mediated individual recognition and prefer to mate with visually familiar males (males that maintain close proximity to females before spawning) [19]. In some other species, social familiarization negatively affects mating preference. For example, female guppies discriminate unfamiliar (novel) males from visually familiarized males and prefer to mate with the unfamiliar male [20]. Mating preference for unfamiliar mates is thought to be important for maintaining high genetic variance in offspring [21]. In contrast, the adaptive significance of mating preference for familiar mates has not been extensively investigated, as there are only limited examples of this preference [22, 23]. Some monogamous rodents, such as prairie voles, prefer to mate with familiar mates, where the formation of a pair bond is important for parental investment [24]. Medaka fish, in contrast, never maintain a monogamous relationship [16] and the adaptive significance of the female mating preference of medaka is unknown.

The behavioral characteristics of male mate-guarding and female mating preference in medaka led us to hypothesize that females become visually familiarized with dominant males that exhibit persistent mate-guarding and that the social familiarization enhances female preference for the dominant males. In this study, we ﻿improved previous behavioral tests [14, 19], which allows males to exhibit mate-guarding while blocking visual familiarization of females with rival males in a triadic relationship. We performed this test using vasotocin receptor V1a2 mutant males that did not exhibit mate-guarding behavior to investigate the relative contribution of proximity and active mate-guarding behavior to the maintenance of mating preferences and male reproductive success. Here we provide results on behaviour involved in triadic interactions that support this hypothesis.

## Results

### Establishment of a novel behavioral test to quantify visual familiarization

**Fig. 1:**
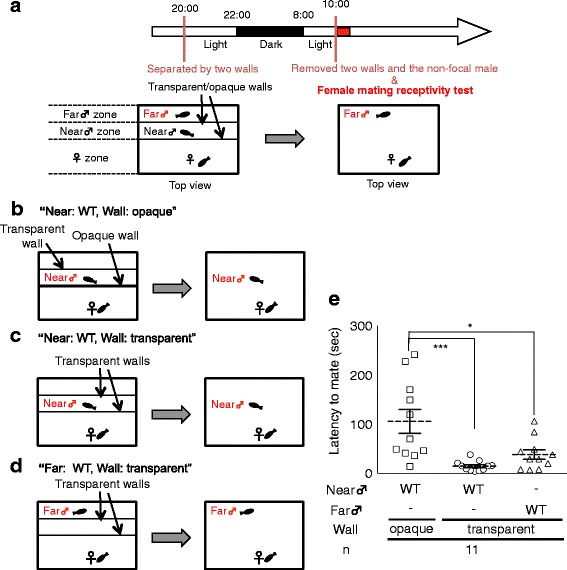
Novel visual familiarization system with separation condition (**a**) Time-course of the female mating receptivity test. A case of far focal male is shown. See the Materials and Methods for details. (**b**-**d**) Separation conditions for the female mating receptivity test in dyadic relationships. Fish were randomly picked from two communal tanks each containing four males and four females and the same males were used several times in the three conditions. (**b**) “Near: WT, Wall: opaque” The female couldn’t see the male in the near zone and mated with it in the next morning. (**c**) “Near: WT, Wall: transparent” The female could see the male in the near zone from a close proximity and mated with it in the next morning. (**d**) “Far: WT, Wall: transparent” The female could see the male in the near zone from some distance and mated with it in the next morning. (**e**) Visual familiarization enhanced female receptivity even if the male was located in the far zone. Mean ± SEM. ***P < 0.0001

To examine whether dominance of persistent mate-guarding enhances familiarization with females while at the same time blocking the female’s familiarization with rival males, we modified the previous behavioral tests [14, 19] and established a novel behavioral test using a tank divided into three zones with two walls (Fig. 1a). First, we placed a female in the larger zone on one side and examined whether the female could become visually familiarized with the wild-type (WT) male in the “far” or “near” zones. To quantify visual familiarization, we performed a female mating receptivity test by calculating the latency to mate with the male of interest, which negatively correlates with female receptivity toward the male. We previously reported that the latency to mate with visually familiarized males is significantly shorter than that with unfamiliar males [19]. One male was placed in either the “far” or the “near” zone, separated by transparent or opaque walls (Fig. 1b-d) in the evening before mating. In this setup, the latency to mate in the opaque wall group (“Near: WT, Wall: opaque”) was significantly longer than that in the transparent wall groups (“Near: WT, Wall: transparent” and “Far: WT, Wall: transparent”; Kruskal-Wallis: chi-squared = 14.931, df = 2, *P* = 0.0005. post-hoc Steel test: “Near: WT, Wall: opaque” VS “Near: WT, Wall: transparent”, *P* = 0.0008; “Near: WT, Wall: opaque” VS “Far: WT, Wall: transparent”, *P* = 0.033; Fig. 1e). Additionally, in the opaque wall group, the number of courtship behaviors (male mating activity) did not significantly decrease, suggesting that low female receptivity toward the male in this group was not derived from decreased male activity (Additional file 1a). These findings indicated that females could become familiarized with males in either the near or the far zone, and confirmed that the female and male could become visually familiarized under this setup.

### Effect of mate-guarding on female visual familiarization

**Fig. 2:**
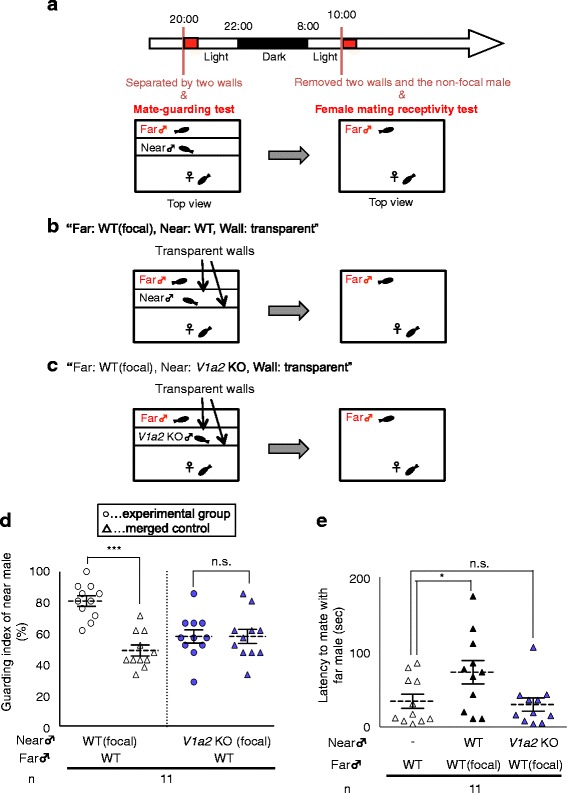
Avoiding enhancement of female receptivity to the subordinate males by mate-guarding. (**a**) Time-course of the mate-guarding test and female mating receptivity test. (**b**-**c**) Separation conditions for the female mating receptivity test in triadic relationships. Fish were randomly picked from two communal tanks each containing four males and four females and the same males were used several times in the three conditions. (**b**) “Far: WT (focal), Near: WT, Wall: transparent”: two WT males were placed in the far and near zones, respectively. The female could see them and mated with the far male in the next morning. (**c**) “Far: WT (focal), Near: *V1a2* KO, Wall: transparent” one WT male and one *V1a2* KO male were placed in the far and near zones, respectively. The female could see them and mated with the far WT male in the next morning. (**d**) Mate-guarding by near males in the separation condition. *V1a2* KO near males didn’t exhibit mate-guarding, whereas WT near males did. The significantly higher guarding indices in the experimental groups compared with those in the merged control groups indicate that near males exhibit mate-guarding. Mean ± SEM. ***P < 0.0001. (**e**) Mate-guarding by the near male suppressed the enhancement of female receptivity to the far male. When *V1a2* KO males were used as the near males, the mean latency to mate with the far males wasn’t significantly different from that when there was no near male. Mean ± SEM. *P < 0.05

Next, we placed the two males and one female in the three zones, which allowed the male in the near zone (near male) to maintain closer proximity to the female than the male in the far zone (far male), and performed a mate-guarding test (Fig. 2a-b). The WT male in the near zone exhibited mate-guarding over the WT male in the far zone (Mann–Whitney *U* test: “WT experimental group” VS “WT negative control”, *U* = 3.50, *N*_1_ = *N*_2_ = 11, *P* < 0.0001; Fig. 2d, Additional file 2). On the following morning, we calculated the latency to mate with the far male in dyadic relationships (Fig. 2a) and found that the presence of the WT male in the near zone significantly decreased female receptivity toward the far male (Kruskal-Wallis: chi-squared = 6.806, df = 2, *P* = 0.0333. post-hoc Steel test: “Far: WT, Wall: transparent” VS “Far: WT (focal), Near: WT, Wall: transparent”, *P* = 0.047; Fig. 2e). In this experimental group, the number of courtship displays was not significantly less than that in control group (Kruskal-Wallis: chi-squared = 1.0216, df = 2, *P* = 0.6. Additional file 1b), confirming that low female receptivity toward the far male was not derived from decreased male activity. These findings indicated that the presence of the near male blocked visual familiarization of the far males. We also investigated whether mate-guarding behavior of the near male was required to block visual familiarization with the far male.

To clarify this issue, we used arginine-vasotocin receptor 2 (*V1a2*) knockout (KO) males generated by TALEN (Transcription Activator-Like Effector Nucleases) methods [25, 26] as the near male (Fig. 2c). Previously, we reported that *V1a2* KO males exhibit defective mate-guarding behavior under free-swimming conditions [14]. The mate-guarding test (Fig. 2a) confirmed that *V1a2* KO near males did not exhibit mate-guarding behavior (Mann–Whitney *U* test: “*V1a2* KO experimental group” VS “*V1a2* KO negative control”, *U* = 55.5, *N*_1_ = *N*_2_ = 11, *P* = 0.759; Fig. 2d, Additional file 3). The *V1a2* KO near male did not maintain its position between the female and the far male, although there was no significant difference in the proximity to the female between the WT near male and the *V1a2* KO near males (Mann–Whitney *U* test: *U* = 57, *N*_1_ = *N*_2_ = 11, *P* = 0.832; Additional file 4). The placement of a *V1a2* KO in the near zone did not affect the latency to mate with the far male (Kruskal-Wallis: chi-squared = 6.806, df = 2, *P* = 0.0333. post-hoc Steel test: “Far: WT, Wall: transparent” VS “Far: WT (focal), Near: *V1a2* KO, Wall: transparent”, *P* = 0.924; Fig. 2e). Additionally, there was no significant difference between the number of courtship display in this group (“Far: WT (focal), Near: *V1a2* KO, Wall: transparent”) and that in control group (“Far: WT, Wall: transparent”) (Kruskal-Wallis: chi-squared = 1.0216, df = 2, *P* = 0.6. Additional file 1b). Furthermore, we confirmed that the free swimming velocity (Mann–Whitney *U* test: *U* = 7, *N*_1_ = *N*_2_ = 5, *P* = 0.310; Additional file 5b) and visual response and locomotion ability (Mann–Whitney *U* test: *U* = 9, *N*_1_ = *N*_2_ = 5, *P* = 0.532; Additional file 5c-d) were normal in mutant males, suggesting that the high female receptivity toward WT far males was not due to abnormal movement of the *V1a2* KO near males. These results demonstrated the necessity of mate-guarding, rather than mere spatial proximity, for inhibiting the formation of familiarity between the female and the rival male. Taken together, our findings indicate that mate-guarding enhanced visual familiarization with the dominant male and blocked the female’s familiarization with the subordinate male, at least under this experimental condition. Furthermore, the presence of the far male did not affect female receptivity to the near male (Mann–Whitney *U* test: *U* = 50, *N*_1_ = *N*_2_ = 11, *P* = 0.507; Additional file 6b). As *V1a2* KO males showed courtship behaviors less frequently than WT males (Mann–Whitney *U* test: *U* = 32.5, *N*_1_ = *N*_2_ = 11, *P* = 0.02; Additional file 7), we could not investigate female receptivity toward the *V1a2* KO males.

### Requirement of persistent mate-guarding for high male mating success

**Fig. 3:**
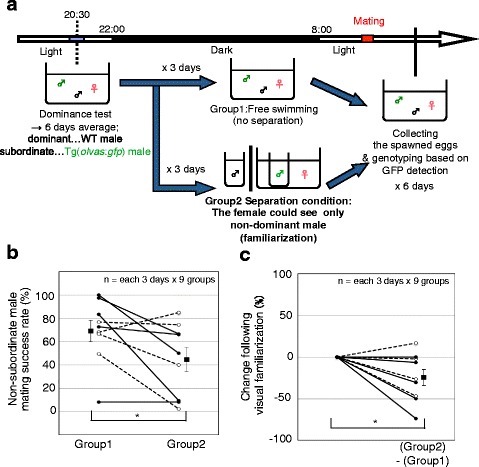
Decreased mating success of non-subordinate males by no visual familiarization with potential mates. (**a**) Procedure for assessing the effect of visual familiarization on the mating success. In the separation condition, the female could see and become familiarized with only the non-dominant male. Based on the 6-day mate-guarding assay, we judged which of the two males was dominant. An example of a dominant WT male is shown. We compared the mean mating success rate of non-subordinate males under normal conditions for 3 days (Group1) and under the separation condition for 3 days (Group2). (**b** and **c**) Decreased mating success of the non-subordinate male by no visual familiarization. Mean ± SEM. *P < 0.05. Solid line and filled circle indicate dominant groups (*N* = 5) and the dashed line and open circle indicate equivalent groups (*N* = 4)

We previously reported that mate-guarding positively correlates with male reproductive success [14]. Here we examined whether visual familiarization is required for the male reproductive success. On the evening before mating, we performed the dominance test and judged which male was dominant (6 successive days; Fig. 3a). The duration of the mate-guarding was significantly different in 5 of 9 groups, while not in 4 groups (the two males were considered to be equivalent). After the dominance test, for 3 of the 6 days, the three fish were allowed to freely swim, whereas on the other 3 days, we added a separation procedure in which the females were visually familiarized with non-dominant males (subordinate males and equivalent males). The next morning, we performed the paternity test and calculated the effect of the separation procedure on the mating success rate (Fig. 3a). The separation procedure significantly decreased the mating success rate of the dominant (*N* = 5) and equivalent (*N* = 4) males (Wilcoxon signed-ranks test: *T* = 3, *N* = 9, *P* = 0.036; Fig. 3b and c). These findings suggest that persistent mate-guarding increased male reproductive success by blocking the familiarization of the female and the rival male, and confirmed the importance of recent familiarization in the development of mating preference in females.

## Discussion

**Fig. 4:**
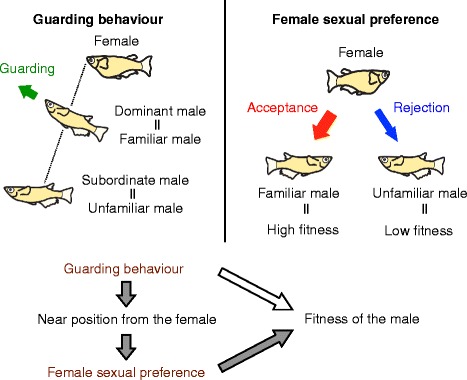
A possible interaction of mate-guarding behavior and female sexual preference in medaka. Persistent mate-guarding behavior leads to high male fitness in medaka, as it allows the dominant male more visual contact with the female than the subordinate male

Mate-guarding had been considered as a type of male-male competition and several studies have demonstrated that mate-guarding increases male fitness by monopolizing the mating opportunities [18, 27]. Many studies have quantified male aggressive behavior towards intruder males as mate-guarding and focused on the interference with a rival male’s approach toward the female, which is one component of mate-guarding. It has remained unclear, however, whether mate-guarding affects male reproductive success [28] via inhibiting the female preference for the rival male. Here we established a novel behavioral test that allows males to exhibit mate-guarding as well as to block visual familiarization of rival males with females in a triadic relationship. Using this setup, we demonstrated that the medaka female likely becomes familiarized with the dominant male during mate-guarding over the subordinate male, leading to a preference of the female for the dominant male (Fig. 4). Although our data could not exclude the possibility that mate-guarding specific effects promote female receptivity, we could not demonstrate a mate-guarding effect on female receptivity in this setup (Additional file 6b). Furthermore, at this time, it is impossible to detect whether mate-guarding promotes female receptivity without familiarization, because the guarding target (female) can see the dominant male, which results in familiarization.

A previous study reported that a medaka female familiarized with two medaka males exhibited almost the same receptivity to both males [19]. In this experiment, two males could not exhibit mate-guarding behavior due to spatial restriction in the small tank. Therefore, it was suggested that familiarity itself was sufficient to generate receptivity. Taken together, although mate-guarding itself may promote female receptivity, it likely enhances female mating preference via promoting familiarization with the female and blocking familiarization between the female and rival male.

In various species, females tend to choose the dominant male as their mating partner [29–32] and this choice provides indirect fitness benefits to females by conferring genetic advantages to the offspring [33]. Thus, in medaka fish, social familiarization may provide opportunities for females to select socially dominant males that win male-male competitions and frequently exhibit mate-guarding. Females may use guarding behavior as a cue indicating the guarding male’s high social dominance status relative to rival males. Furthermore, considering that many fish species avoid mating or grouping with parasitic individuals [34], female mating preference for familiar males may allow female medaka to prevent mating with possibly infected unfamiliar males and to mate with familiar males whose health has been assessed by the female during familiarization.

These findings will enhance our understanding of the adaptive benefit of persistent mate-guarding. Although persistent mate-guarding is reported in monogamous species in which parental care is necessary for offspring growth [24], medaka fish are not monogamous. Here we demonstrated that a mutant near male that did not exhibit mate-guarding, but maintained close proximity to the female, did not block the visual familiarization of the female with the far male (Fig. 2e). Furthermore, visual familiarization between the female and male was required for maintaining the mating success rate (Fig. 3). Our laboratory experiments suggest that persistent mate-guarding allows dominant males to increase their fitness by enhancing the preference of the female for the dominant male in medaka fish. Mate-guarding in some internal fertilization species is considered to be an adaptive behavior that minimizes extra-pair copulation during female fertile periods. In these species, the last male to mate with the female tends to have the highest reproductive success, as males can remove the sperm of former males by the ejaculation of new sperm [35, 36]. In contrast, in external fertilization species like medaka fish [19], the first male to induce female spawning has the advantage of producing offspring, as a high probability of fertilization results from the close proximity of males to females in ejaculation and the timing of ejaculation coordinated with female egg release [37]. Furthermore, in some fish species the females are able to determine the timing of their spawning. For example, medaka females spawn only when they accept courtship from a preferred male and otherwise reject spawning by escaping from the males [19]. Taken together, being selected by a female as a mating partner might be more important for a high probability of fertilization in medaka than in internal fertilization species.

Our studies suggest that females prefer to mate with males that dominantly exhibit mate-guarding behavior. Future studies should investigate whether similar interactions between female preference and mate-guarding occur in other species, particularly monogamous species, because some monogamous species exhibit both mate-guarding and preference for familiar mates [23, 38, 39]. For example, prairie voles exhibit agonistic behavior toward non-mates (mate-guarding), while they show affiliation toward a mate [39]. These behaviors have only been described in dyadic setups, however, and whether mate-guarding activates preference for mates and blocks pair-bonding with non-mates is unknown. Further comparative studies are required to elucidate the interaction of female mating preference and male-male competition in the behavioral interactions underlying social bonding via individual recognition. In addition, as the present study was performed under laboratory conditions using a laboratory bred strain, ecological studies using natural populations under natural conditions will be essential to further elucidate the adaptive and evolutionary significance of the interaction between male-male competition and female mating preference.

## Conclusions

Although male-male competition and female mating preference have been investigated in various animals individually, the interaction between them is largely unknown. Here we found that mate-guarding, not simply close proximity, led to familiarization with the female while at the same time blocking the female’s visual familiarization with the other male in medaka fish. Thus some behavioral component of mate-guarding is likely to increase the salience of the male’s appearance to facilitate familiarization. In addition, we found that persistent mate-guarding until spawning was required for high mating success. These findings suggested that mate-guarding allows males to gain familiarity with the female over their rivals, which may enhance female preference for the dominant male.

## Methods

### Ethical note

The work in this paper was conducted using protocols specifically approved by the Animal Care and Use Committee of the University of Tokyo (permit number: 12–07). All efforts were made to minimize suffering, following the NIH Guide for the Care and Use of Laboratory Animals.

### Fish and breeding conditions

Medaka fish were maintained in groups in plastic aquariums (13 cm x 19 cm x 12 cm [height]). All fish were hatched and bred in our laboratory. Sexually mature male (1.7〜2.6 cm) and female (1.8〜2.4 cm) medaka 3 ~ 5 months of age producing fertilized eggs every morning were used. The water temperature was ~28 °C and light was provided by standard fluorescent lamps for 14 h per day (08:00–22:00).

### Female mating receptivity test

To quantify the motivation of a female to mate with a male of interest, a female mating receptivity assay was performed as previously described with minor modifications [19]. Fish were randomly picked from two communal tanks each containing four males and four females. On the day before the assay, “one male and one female (Figs. 1b-d)” or “two males and one female (Figs. 2b-c)” were placed in a tank and then separated in the evening (20:00-21:00) by two walls to create a female zone, a near zone including one of the males, and a far zone including the second male (“female zone”: 6.5 cm x 19 cm x 12 cm [height], “near zone” and “far zone”: each 3.25 cm x 19 cm x 12 cm [height]) (Fig. 1a). These walls could either be transparent or opaque to allow us to examine the effect of familiarization on female mating receptivity. The next morning (10:00-12:00) the two walls (Figs. 1b-d, 2b-c) and the non-focal male (Figs. 2b-c) were removed from the tank and only the focal male was left with the female. Mating behavior was recorded for 5 min. Based on the recording, the timing of the male quick-circle courtship displays and copulations followed by spawning by the pair were determined. We compared the interval between the first male courtship and the first mating (latency to mate) in each group. The latency to mate negatively correlates with female mating receptivity. We confirmed the normal male mating activity in each group by comparing the number of courtships (Additional file 1, Additional file 6c). The same trios were used for female receptivity tests and mate-guarding tests.

### Mate-guarding test

A mate-guarding test to evaluate whether males exhibited mate-guarding in individual experimental conditions was performed as previously described with minor modifications [14]. Fish were randomly picked from two communal tanks each containing four males and four females. One female and two males were placed separately in an aquarium divided into three zones (Fig. 2a) by two transparent walls in the same way as the female mating receptivity test,, and their behavior was recorded from the bottom of the aquarium in the evening (20:00 to 21:00). All male pairs were size-matched. As a negative control group (merged group), we performed the same experiment using virtually merged trios, recording one female and two males one by one, each placed in a separate aquarium. We converted video files into 21 image sequences per 5 s, and manually spotted the head and tail positions of the three medaka fish using ImageJ (NIH, Bethesda, MD, USA) to calculate the center positions as the body positions. Based on the positions of the female (x_F_, y_F_), the male in the far zone (far male) (x_Mf_, y_Mf_), and the male in the near zone (near male) (x_Mn_, y_Mn_), the relative positions of the near male (X, Y) were calculated when the female and far male positions were defined as (0, 0) and (1, 0), respectively. We spotted the relative positions of the near male and defined a circle with center (1/2, 0) and radius 1/2 as the “guarding circle”. When the near male was within the guarding circle, the angle between the vectors from the near male to the female and from the near male to the far male was obtuse. The probability of being in the guarding circle was defined as the “guarding index”. The significantly higher guarding indices in the experimental groups compared with those in the merged groups indicate that the near males in the experimental groups exhibit mate-guarding. The next morning (10:00-12:00) the two walls and one of the males (i.e. non-focal male; either near or far male) were removed from the tank and the other male was left with the female, and the female mating receptivity test was performed.

### Visual response and locomotion ability test (Optomotor response)

An optomotor response test to check the visual response and locomotion ability was performed as previously described [40] with minor modification. The apparatus is shown in Additional file 5a. The medaka were placed in a fixed 15-cm-diameter circular tank with a water depth of 2 cm. A striped cylinder positioned on a rotatable metal disk driven by a motor. At first, the fish was transferred into the circular tank and adapted to the apparatus for ~1 min. Next, we recorded free-swimming for 1 min to calculate the velocity. Finally, the optomotor response was recorded for 1 min after adaptation to the cylinder rotation. A series of frames was analyzed using the software UMATracker (http://ymnk13.github.io/UMATracker/).

### Dominance test

A dominance test to determine which male is dominant in mate-guarding was performed as previously described [14]. We used one WT male and one transgenic (*homozygote olvas*:*gfp*) male and compared the degree of mate-guarding behavior in the presence of a female. All male pairs were size-matched. We measured the relative locations of the three fish and calculated the probability of the WT male being in the guarding circle when the female and transgenic (*homozygote olvas*:*gfp*) male positions were defined as (0, 0) and (1, 0), respectively. We defined this probability as the “guarding index of WT males”. We also calculated the probability of the transgenic (*homozygote olvas*:*gfp*) male being in the guarding circle when the female and WT male positions were defined as (0, 0) and (1, 0), respectively. We defined this probability as the “guarding index of transgenic (*homozygote olvas*:*gfp*) males” and compared this index with that of WT. A higher guarding index indicates higher dominance in the mate-guarding behavior compared with the other male. We performed this dominance test for 6 days using the same 3 fish (6 trials) and compared the average guarding index of the two males for 6 days. In 5/9 groups, either the WT or transgenic male was dominant. In 4/9 groups, they were equal (i.e., no significant difference between their mean guarding indices. Mann–Whitney U test: *P* > 0.05).

### Paternity test

After the dominance test, a paternity test was performed as previously described [14] with minor modification (Fig. 3a) using a separate procedure to examine whether visual familiarization is required for dominant males to maintain their high mating success rate. After the dominance test, for 3 of the 6 days, the 3 fish were allowed to freely swim in the same tank without any procedure until mating. On the other 3 days, we added a separation procedure in which the females were visually familiarized with non-dominant males (subordinate males and equal males), i.e., the males that did not dominantly exhibit mate-guarding, until mating. The next morning, we collected fertilized eggs from the female and genotyped the progeny. Transgenic fish were distinguished from WT fish by the GFP fluorescence of the primordial germ cells. Using this method, we measured the mating success rate of the non-subordinate males (dominant males and equal males) and evaluated the effect of the separation procedure on the mating success rate.

### Statistical analysis

To examine whether visual contact affects female mating receptivity to males and whether mate-guarding by near males affects female mating receptivity to far males, we compared the latency to mate between each experimental group and that of the control group using the Kruskal-Wallis test (post-hoc Steel test) implemented in EZR (Saitama Medical Center, Jichi Medical University, Saitama, Japan). To determine whether WT males and *V1a2* KO males exhibited mate-guarding (mate-guarding test), we compared the guarding index of males of each genotype in the experimental groups with that of the negative control using a Mann–Whitney U test implemented in Prism 6 (GraphPad). Furthermore, to examine other behavioral phenotypes of the *V1a2* KO males, the free-swimming velocity, the ratio of fish angular speed to strip speed in the optomotor response test, and the courtship frequency of *V1a2* KO males were compared with those of WT males using a Mann–Whitney U test implemented in Prism 6 (GraphPad). In the dominance test, we compared the guarding index of two males for 6 days using the Mann–Whitney U test implemented in Prism 6 (GraphPad) and judged which male was dominant in each group. To analyze the effect of visual familiarization on the mating success rate, we compared the non-subordinate male mating success rate in the no separation group with that in the separation group using the Wilcoxon signed-ranks test implemented in Prism 6 (GraphPad). All p values are two-tailed.

### Availability of data and materials

The datasets supporting the conclusions of this article are included within the article and its additional files.

## Additional files

Additional file 1: The normal male mating activity in the female mating receptivity test. (a) The visual familiarization didn’t significantly affect the motivation to mate of males. Mean ± SEM. Kruskal-Wallis: chi-squared = 6.686, df = 2, *P* = 0.0353. post-hoc Steel test: “Near: WT, Wall: opaque” VS “Near: WT, Wall: transparent”, *P* = 0.115; “Near: WT, Wall: opaque” VS “Far: WT, Wall: transparent”, *P* = 0.689. (b) The existence of the near male didn’t significantly affect the motivation to mate of the far male. Mean ± SEM. Kruskal-Wallis: chi-squared = 1.0216, df = 2, *P* = 0.6. (PDF 106 kb) Additional file 2: Mate-guarding movie of the WT male in the separate condition. The WT near male maintains its position between the female and the far male even in the separate condition. This movie is played at quadruple speed. (MOV 39999 kb) Additional file 3: Mate-guarding movie of the *V1a2* KO male in the separate condition. The *V1a2* KO near male didn’t maintain its position between the female and the far male. This movie is played at quadruple speed. (MOV 35412 kb) Additional file 4: Distance between the female and the near male under the separation condition. There was no significant difference in the distance between the female and the WT near male or the *V1a2* KO near males in mate-guarding test. Mean ± SEM. (PDF 58 kb) Additional file 5: No significant defect in visual capacity or locomotion in *V1a2* KO males. (a) Apparatus for analysis of the optomotor response described previously [40]. (b) The moving velocity for 1 min of free-swimming *V1a2* KO males did not differ significantly from that of WT males. Mean ± SEM. Mann–Whitney *U* test: *U* = 7, *N*_1_ = *N*_2_ = 5, *P* = 0.310. (c-d) Integrated angular velocity during 1 min of free-swimming in (c) WT and (d) *V1a2* KO males. Each line indicates the raw data of five individual fish. (e) Ratio of the mean fish angular speed to mean stripe speed. Mean ± SEM. Mann–Whitney *U* test: *U* = 9, *N*_1_ = *N*_2_ = 5, *P* = 0.532. (PDF 197 kb) Additional file 6: No significant effect of the presence of a far male to the female receptivity. (a) A Separation condition for the female mating receptivity test in triadic relationships. “Far: WT, Near: WT (focal), Wall: transparent”: two WT males were placed in the far and near zones, respectively. The female could see them and mated with the near male in the next morning. (b) The presence of a far male didn’t affect the female receptivity to the near male. Mean ± SEM. (c) The existence of the far male didn’t significantly affect the motivation to mate of the near male. Mean ± SEM. Mann–Whitney *U* test: *U* = 39, *N*_1_ = *N*_2_ = 11, *P* = 0.161. (PDF 109 kb) Additional file 7: Low motivation for courtship behavior in *V1a2* KO males. *V1a2* KO males exhibited courtship behavior less frequently than WT males. Mean ± SEM. Mann–Whitney *U* test: *U* = 32.5, *N*_1_ = *N*_2_ = 11, *P* = 0.02. (PDF 62 kb)

## References

[1] Hunt J, Breuker CJ, Sadowski JA, Moore AJ (2009). Male-male competition, female mate choice and their interaction: determining total sexual selection. J Evol Biol..

[2] Paull GC, Filby AL, Giddins HG, Coe TS, Hamilton PB, Tyler CR (2010). Dominance hierarchies in zebrafish (Danio rerio) and their relationship with reproductive success. Zebrafish..

[3] Botero CA, Rossman RJ, Caro LM, Stenzier LM, Lovette IJ, De Kort SR (2009). Syllable Type Consistency is Related to Age, Social Status, and Reproductive Success in the Tropical Mockingbird. Anim Behav..

[4] Rodriguez-Llanes JM, Verbeke G, Finlayson C (2009). Reproductive benefits of high social status in male macaques (*Macaca*). Anim Behav..

[5] Andersson M (1982). Female choice selects for extreme tail length in a widowbird. Nature..

[6] Kodric-Brown A, Nicoletto PF (2001). Female choice in the guppy (*Poecilia reticulate*): the interaction between male color and display. Behav Ecol Sociobiol..

[7] Wagner WEJ, Reiser MG (2000). The importance of calling song and courtship song in females mate choice in the variable field cricket. Anim Behav..

[8] Birkhead TR (1979). Mate guarding in the magpie *Pica pica*. Anim Behav..

[9] Calbacho-Rosa L, Córdoba-Aguilar A, Peretti AV (2010). Occurrence and duration of post-copulatory mate guarding in a spider with last sperm precedence. Behavior..

[10] Miki M (2003). Costs of mate guarding and opportunistic mating among wild male Japanese macaques. Int J Primatol.

[11] Parker GA (1970). Sperm competition and its evolutionary consequence in the insects. Biol Rev..

[12] Robinson SK (1986). Benefits, costs, and determinants of dominance in a polygynous oriole. Anim Behav..

[13] Schubert M, Schradin C, Toedel HG, Pillay N, Ribble DO (2009). Male mate guarding in a socially monogamous mammal, the round-eared sengi: on costs and trade-offs. Behav Ecol Sociobiol..

[14] Yokoi S, Okuyama T, Kamei Y, Naruse K, Taniguchi Y, Ansai S (2015). An essential role of the arginine vasotocin system in mate-guarding behaviors in triadic relationships of medaka fish (*Oryzias latipes*). PLoS Genet..

[15] Fukamachi S, Kinoshita M, Aizawa K, Oda S, Meyer A, Mitani H (2009). Dual control by a single gene of secondary sexual characters and mating preferences in medaka. BMC Biol..

[16] Kobayashi M, Yoritsune T, Suzuki S, Shimizu A, Koido A, Kawaguchi Y (2012). Reproductive behavior of wild medaka in an outdoor pond. Nippon Suisan Gakkaishi..

[17] Ono Y, Uematsu T (1957). Mating ethogram in *Oryzias latipes*. J Fac Sci Hokkaido Univ..

[18] Parker GA (1974). Courtship persistence and female-guarding as male time investment strategies. Behavior..

[19] Okuyama T, Yokoi S, Abe H, Isoe Y, Suehiro Y, Imada H (2014). A neural mechanism underlying mating preference for familiar individuals in medaka fish. Science..

[20] Hughes KA, Du L, Rodd FH, Reznick DN (1999). Familiarity leads to female mate preference for novel males in the guppy, *Poecilia reticulata*. Anim Behav.

[21] Penn D, Potts W (1998). MHC-disassortative mating preferences reversed by cross-fostering. Proc Biol Sci..

[22] Cheetham SA, Thom MD, Beynon RJ, Hurst JL (2008). The effect of familiarity on mate choice.

[23] Senar JC, Mateos-Gonzalez F, Urive F, Arroyo L (2013). Familiarity adds to attractiveness in matters of siskin mate choice. Proc Biol Sci..

[24] Shapiro LE, Austin D, Ward SE, Dewsbury DA (1986). Familiarity and female mate choice in 2 species of voles (*Microtus-Ochrogaster* and *Microtus-Montanus*). Anim Behav..

[25] Ansai S, Sakuma T, Yamamoto T, Ariga H, Uemura N, Takahashi R (2013). Efficient targeted mutagenesis in medaka using custom-designed transcription activator-like effector nucleases. Genetics..

[26] Ansai S, Inohaya K, Yoshiura Y, Schartl M, Uemura N, Takahashi R (2014). Design, evaluation, and screening methods for efficient targeted mutagenesis with transcription activator-like effector nucleases in medaka. Dev Growth Differ..

[27] Engelhardt A, Heistermann M, Hodges JK, Nürnberg P, Niemitz C (2006). Determinants of male reproductive success in wild long-tailed macaques (*Macaca fascicularis*)-male monopolisation, female mate choice or post-copulatory mechanisms?. Behav Ecol Sociobiol..

[28] Wong BB, Candolin U (2005). How is female mate choice affected by male competition?. Biol Rev Camb Philos Soc..

[29] Fisher HS, Swaisgood R, Fitch-Snyder H (2003). Countermarking by male pygmy lorises (Nycticebus pygmaeus):do females use odor cues to select mates with high competitive ability?. Behav Ecol Sociobiol..

[30] Mateos C, Carranza J (1999). Effects of male dominance and courtship display on female choice in the ring-necked pheasant. Behav Ecol Sociobiol..

[31] Nilsson SÖ, Nilsson GE (2000). Free choice by female sticklebacks: lack of preference for male dominance traits. Can J Zool..

[32] Rich TJ, Hurst JL (1998). Scent marks as reliable signals of the competitive ability of mates. Anim Behav..

[33] Moore AJ (1994). Genetic evidence for the “good genes” process of sexual selection. Behav Ecol Sociobiol..

[34] Barber I, Hoare D, Krause J (2000). Effects of parasites on fish behavior: a review and evolutionary perspective. Rev Fish Biol Fish..

[35] Birkhead TR, Martínez JG, Burke T, Froman DP (1999). Sperm mobility determines the outcome of sperm competition in the domestic fowl. Proc Biol Sci..

[36] Parker GA, Blum MS, Blum NA (1979). Sexual selection and sexual conflict. Sexual selection and reproductive competition in insects.

[37] Stoltz JA, Neff BD (2006). Sperm competition in a fish with external fertilization: the contribution of sperm number, speed and length. J Evol Biol..

[38] Lim MM, Wang Z, Olazabal DE, Ren X, Terwilliger EF, Young LJ (2004). Enhanced partner preference in a promiscuous species by manipulating the expression of a single gene. Nature..

[39] McGraw LA, Young LJ (2010). The prairie vole: an emerging model organism for understanding the social brain. Trends Neurosci..

[40] Imada H, Hoki M, Suehiro Y, Okuyama T, Kurabayashi D, Shimada A (2010). Coordinated and cohesive movement of two small conspecific fish induced by eliciting a simultaneous optomotor response. PLoS One.

