# Supplementary figures and images for: Mate-guarding behavior enhances male reproductive success via familiarization with mating partners in medaka fish
Source: Front Zool. 2016 Jun 2;13:21. doi: 10.1186/s12983-016-0152-2 (PMC4890520; doi:10.1186/s12983-016-0152-2)

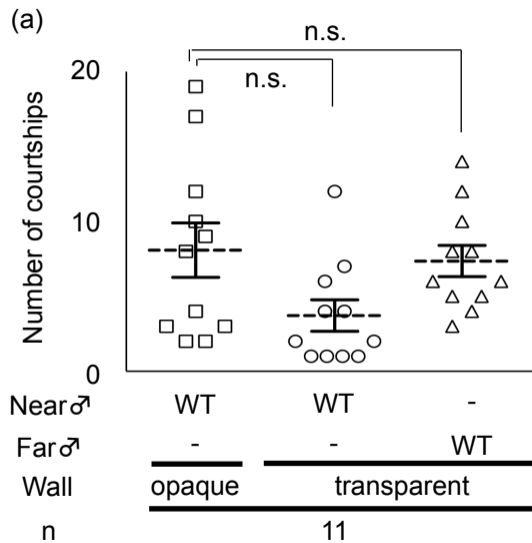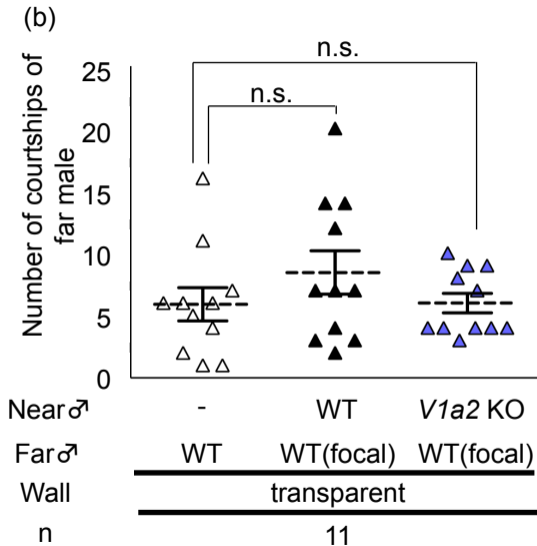

Supplement: Additional file 1: — The normal male mating activity in the female mating receptivity test. (a) The visual familiarization didn’t significantly affect the motivation to mate of males. Mean ± SEM. Kruskal-Wallis: chi-squared = 6.686, df = 2, P = 0.0353. post-hoc Steel test: “Near: WT, Wall: opaque” VS “Near: WT, Wall: transparent”, P = 0.115; “Near: WT, Wall: opaque” VS “Far: WT, Wall: transparent”, P = 0.689. (b) The existence of the near male didn’t significantly affect the motivation to mate of the far male. Mean ± SEM. Kruskal-Wallis: chi-squared = 1.0216, df = 2, P = 0.6. (PDF 106 kb) [file 12983_2016_152_MOESM1_ESM.pdf]

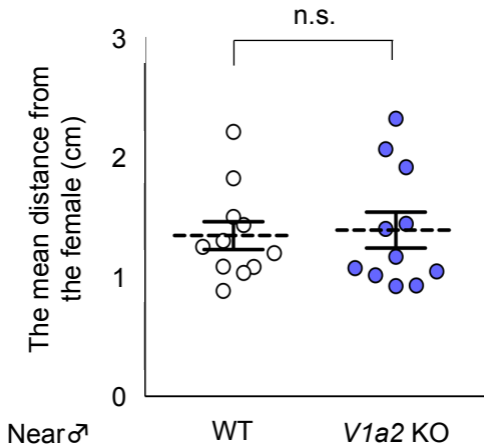

Supplement: Additional file 4: — Distance between the female and the near male under the separation condition. There was no significant difference in the distance between the female and the WT near male or the V1a2 KO near males in mate-guarding test. Mean ± SEM. (PDF 58 kb) [file 12983_2016_152_MOESM4_ESM.pdf]

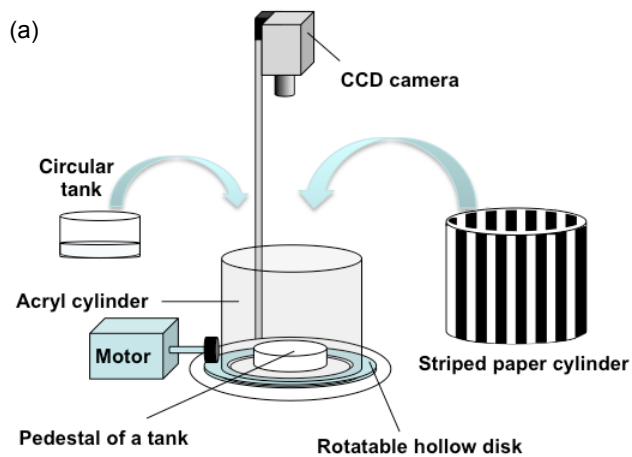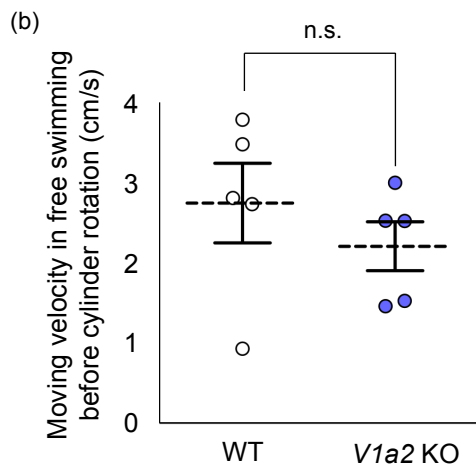

( $\times 10^3$  degree)

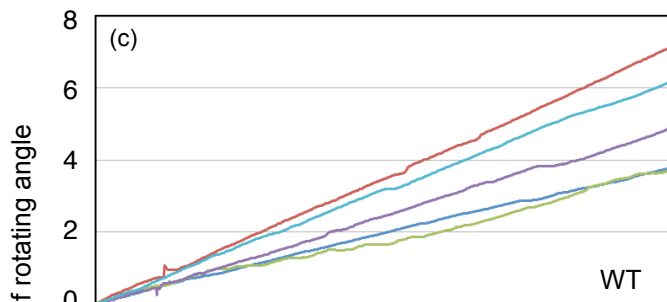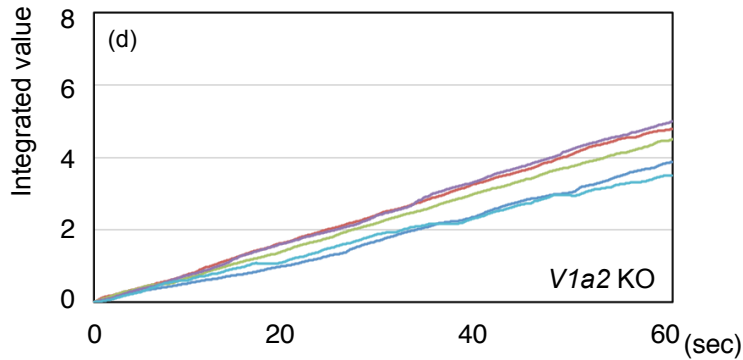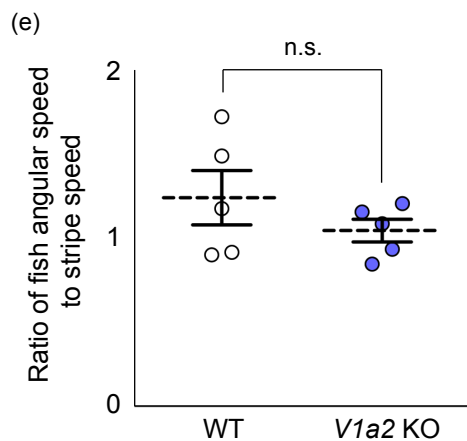

Supplement: Additional file 5: — No significant defect in visual capacity or locomotion in V1a2 KO males. (a) Apparatus for analysis of the optomotor response described previously [40]. (b) The moving velocity for 1 min of free-swimming V1a2 KO males did not differ significantly from that of WT males. Mean ± SEM. Mann–Whitney U test: U = 7, N 1 = N 2 = 5, P = 0.310. (c-d) Integrated angular velocity during 1 min of free-swimming in (c) WT and (d) V1a2 KO males. Each line indicates the raw data of five individual fish. (e) Ratio of the mean fish angular speed to mean stripe speed. Mean ± SEM. Mann–Whitney U test: U = 9, N 1 = N 2 = 5, P = 0.532. (PDF 197 kb) [file 12983_2016_152_MOESM5_ESM.pdf]

(a) “Far ... WT, Near ... WT(focal), wall ... transparent”

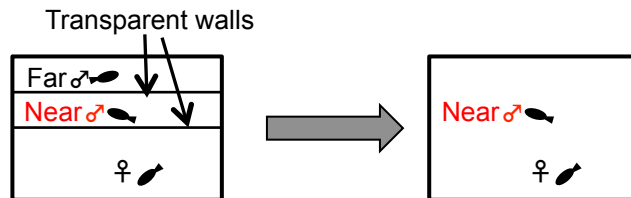

(b)

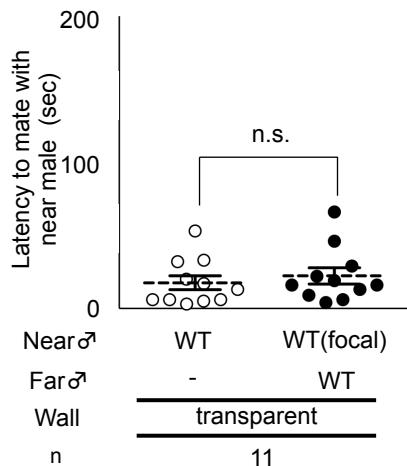

(c)

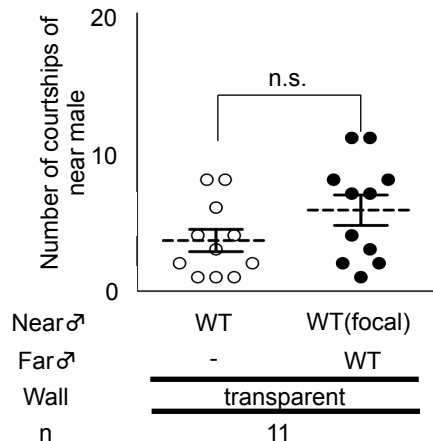

Supplement: Additional file 6: — No significant effect of the presence of a far male to the female receptivity. (a) A Separation condition for the female mating receptivity test in triadic relationships. “Far: WT, Near: WT (focal), Wall: transparent”: two WT males were placed in the far and near zones, respectively. The female could see them and mated with the near male in the next morning. (b) The presence of a far male didn’t affect the female receptivity to the near male. Mean ± SEM. (c) The existence of the far male didn’t significantly affect the motivation to mate of the near male. Mean ± SEM. Mann–Whitney U test: U = 39, N 1 = N 2 = 11, P = 0.161. (PDF 109 kb) [file 12983_2016_152_MOESM6_ESM.pdf]

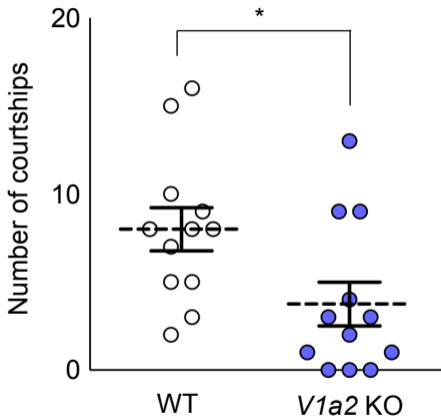

Supplement: Additional file 7: — Low motivation for courtship behavior in V1a2 KO males. V1a2 KO males exhibited courtship behavior less frequently than WT males. Mean ± SEM. Mann–Whitney U test: U = 32.5, N 1 = N 2 = 11, P = 0.02. (PDF 62 kb) [file 12983_2016_152_MOESM7_ESM.pdf]
